# Successful treatment of severe renal failure caused by malignant hypertension using a combination of renin–angiotensin–aldosterone system inhibitors: a case report

**DOI:** 10.1007/s13730-024-00934-7

**Published:** 2024-09-30

**Authors:** Wataru Harada, Yujiro Maeoka, Akira Takahashi, Mahoko Yoshida, Yosuke Osaki, Naoki Ishiuchi, Kensuke Sasaki, Takao Masaki

**Affiliations:** Department of Nephrology, Hiroshima University Hospital, 1-2-3 Kasumi, Minami-Ku, Hiroshima, 734-8551 Japan

**Keywords:** Malignant hypertension, Mineralocorticoid receptor antagonist, Direct renin inhibitor, Renin–angiotensin–aldosterone system

## Abstract

Marked activation of the renin–angiotensin–aldosterone system (RAAS) plays an important role in malignant hypertension (MHT) by worsening hypertension and renal function. The rates of readmission for severe hypertension and cardiovascular disease in such emergencies are high, suggesting that suppression of the RAAS may be inadequate during the acute phase in some cases. This report presents a case of MHT complicated with renal insufficiency (creatinine 3.93 mg/dL) and massive proteinuria, in which antihypertensive therapy, including an angiotensin receptor blocker, aliskiren, and spironolactone, normalized blood pressure (BP) and preserved renal function. Plasma renin activity was extremely high (131.9 ng/mL/h) on admission but normalized within almost 2 weeks. Although aliskiren and spironolactone were discontinued before discharge, BP was well controlled and renal function was further improved (creatinine 1.14 mg/dL) at follow-up 24 months later. This case of renal failure induced by MHT was successfully treated with a combination of RAAS inhibitors during the acute phase. The controlled BP and improved renal function in this patient suggest that adequate suppression of the RAAS cascade during the acute phase is potentially effective in terms of breaking the vicious cycle of MHT with hyperreninemia.

## Introduction

Malignant hypertension (MHT) is a type of hypertensive emergency that is characterized by severe spikes in blood pressure (BP) and acute microvascular injury to various organs, including the kidney [1]. Although advances in the development of antihypertensive medication have improved BP control [2], a recent study showed that the 30-day readmission rate for acute severe hypertension, heart failure, or stroke following hospitalization for hypertensive emergency is still high [3]. Therefore, BP may remain uncontrolled during the acute phase of a hypertensive emergency in some patients, leading to readmission.

Marked activation of the renin–angiotensin–aldosterone system (RAAS) is crucial in the development of MHT [1]. In these conditions, severe hypertension induces microvascular damage and pressure natriuresis, leading to ischemia of the renovascular bed and paradoxical activation of the RAAS. Inappropriate renin secretion, leading to high levels of angiotensin II and aldosterone, facilitates vasoconstriction and impairs urinary sodium excretion, accelerating hypertension and renal dysfunction [1, 2]. Inhibition by angiotensin-converting enzyme inhibitors (ACEIs) or angiotensin receptor blockers (ARBs) has been proposed to be a potentially effective treatment strategy in patients with pathophysiological activation of the RAAS [4]. These findings suggest that the RAAS plays a significant role in MHT and that use of renin–angiotensin–aldosterone system inhibitors (RASIs) may have potential benefits in terms of improving renal function by breaking the vicious cycle of MHT.

The incidence of hyperkalemia and hypotension in diabetic kidney disease is higher when combining an ARB or ACEI with aliskiren or spironolactone compared to using an ARB or ACEI alone, although albuminuria is decreased with the combination [5, 6]. Given that hypokalemia and severe hypertension resulting from marked activation of the RAAS are frequently observed during the acute phase in patients with MHT, it is conceivable that the adverse effects of RAAS combination therapy are beneficial in these patients. However, there is limited research on the effects of direct renin inhibitors (DRIs) and mineralocorticoid receptor antagonists (MRAs) in patients with MHT and few relevant reports [7, 8]. Moreover, although there have been some reports on use of dual RASI therapy (ACEI + ARB or ARB + MRA) [8–11], to the best our knowledge, there have been none on triple RASI therapy (DRI + MRA + ARB). This report describes a case in which BP was successfully controlled by a combination of a DRI, MRA, and ARB during the acute phase of MHT, leading to recovery of renal function.

## Case report

A 41 year-old woman with no history of hypertension visited a local doctor because of deteriorating eyesight. She was an ex-smoker of up to 10 cigarettes per day but did not take any medications. She was diagnosed to have severe hypertension, renal dysfunction and referred to our hospital.

On admission, her BP was alarmingly high at 242/186 mmHg, and her pulse rate was 111 beats per minute. Her other vital signs were normal. Physical examination revealed no significant findings apart from deterioration of her eyesight. Her consciousness was clear, and there were no signs of convulsions or involuntary movements. Laboratory data indicated renal failure with proteinuria (creatinine 3.93 mg/dL, proteinuria 4.0 g/day), hypokalemia (potassium 2.7 mEq/L), and secondary hyperaldosteronism as a result of excessive renin activity (131.9 ng/mL/h). Her lactate dehydrogenase (LDH) level was elevated at 847 U/L. Abdominal ultrasonography showed that both kidneys were of normal size. A retinal examination revealed papilledema. She was diagnosed to have MHT with renal failure.

**Fig. 1:**
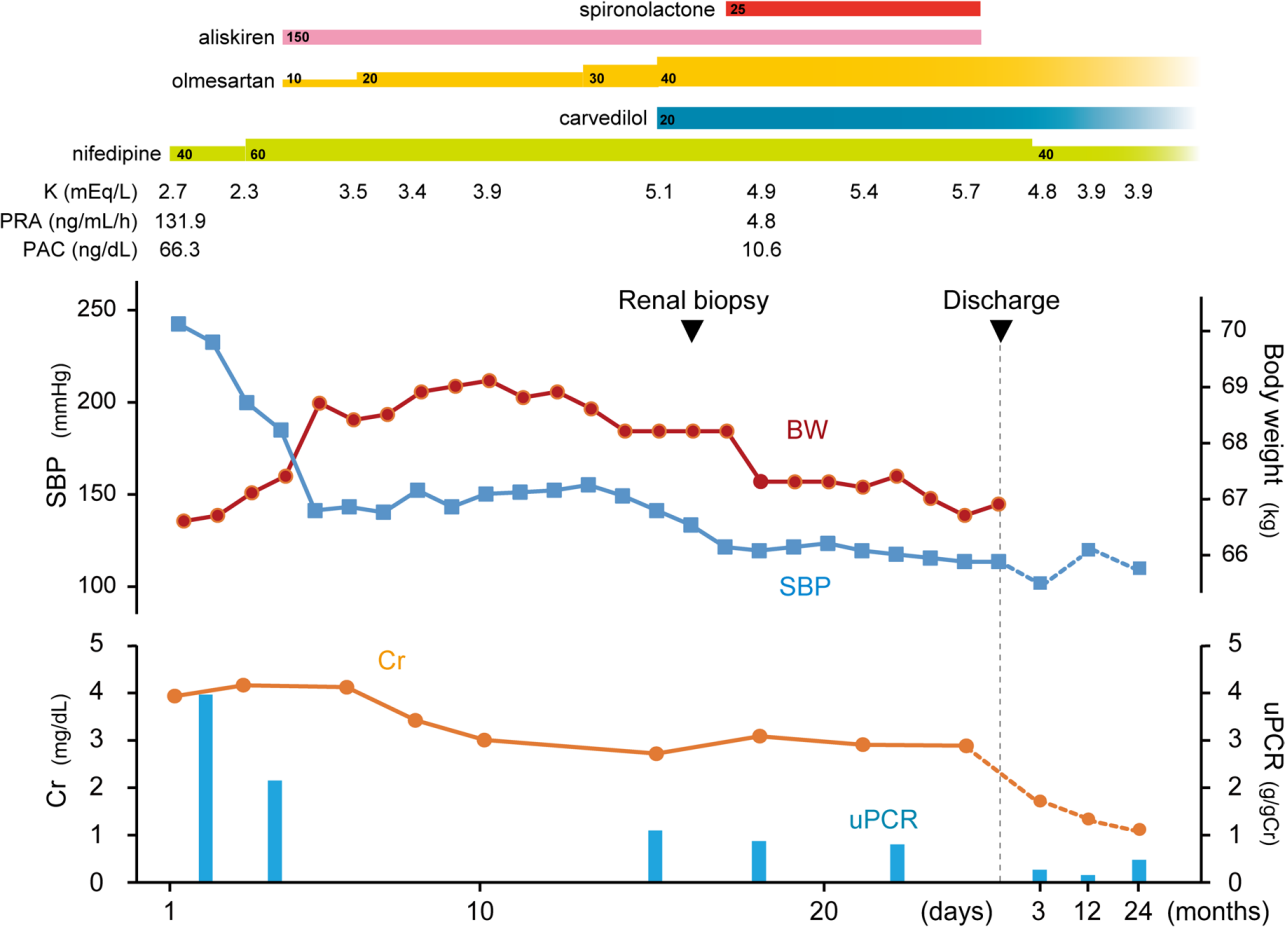
Time course of the patient’s clinical illness. Changes in systolic blood pressure (SBP, blue line), body weight (BW, red line), serum creatinine (Cr, orange line), and the urine protein/creatinine ratio (uPCR, blue bars). PAC, plasma aldosterone concentration; PRA, plasma renin activity

She was started on oral antihypertensive treatment consisting of a calcium channel blocker (CCB) and potassium supplementation immediately after admission. The CCB was increased to the maximum dosage on hospital day 3. Considering that magnetic resonance angiography revealed no stenotic lesions in the renal arteries, aliskiren 150 mg/day and a low dose of an ARB were added on hospital day 4, after which the ARB dosage was gradually increased to the maximum, taking care to avoid excessive hypotension and deterioration of renal function. Within one week, her BP improved significantly from 242/186 mmHg to approximately 150/90 mmHg, with a decrease in both the serum creatinine level and proteinuria (Fig. 1).

**Fig. 2:**
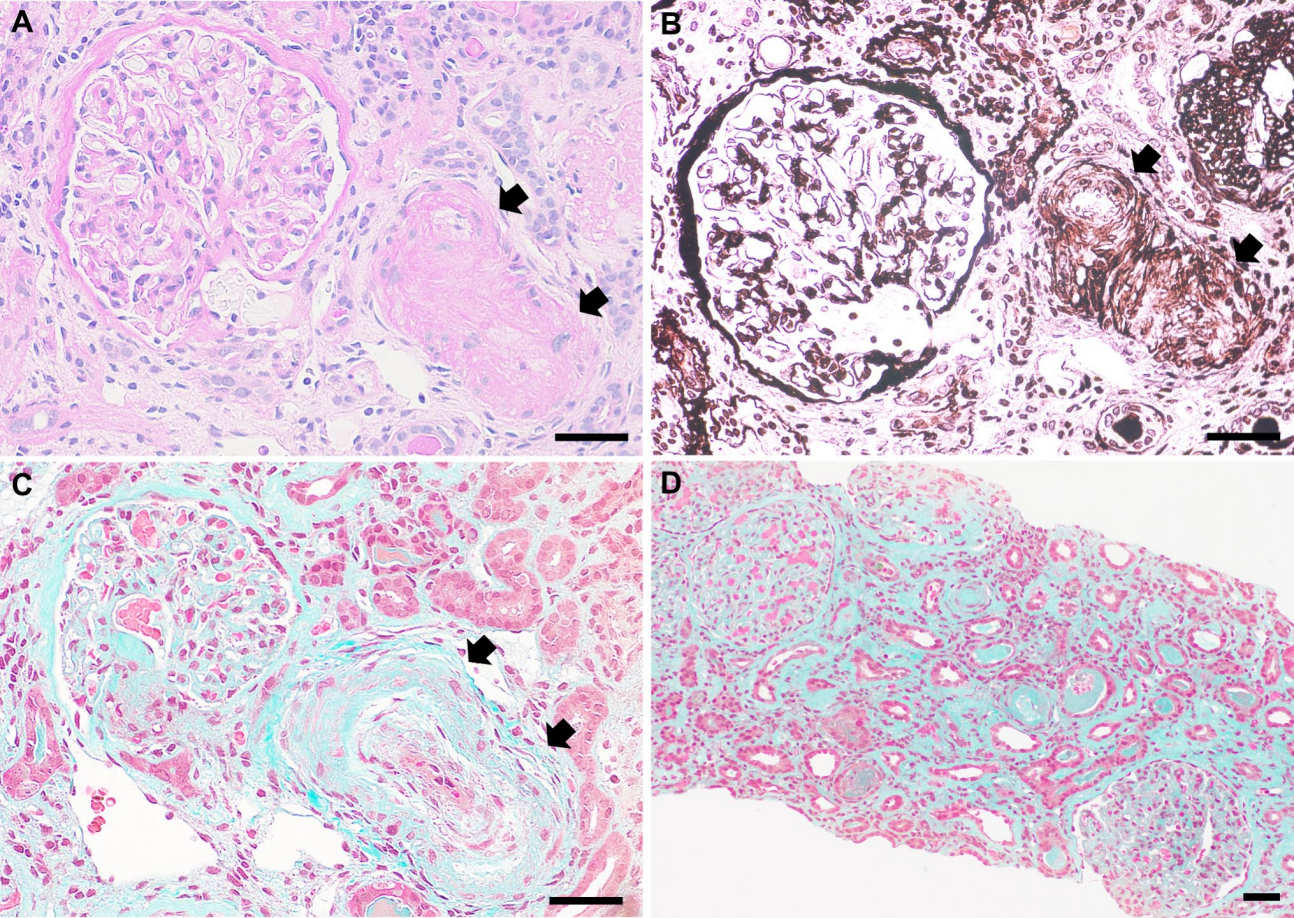
Findings on renal pathological examination. Light microscopic views of small renal arterial occlusive (onion skin-like) lesions (*arrows*) under periodic acid-Schiff **A**, periodic acid-methenamine silver **B**, and Masson’s trichrome **C** staining. Masson’s trichrome staining also showed atrophic changes in the tubules and fibrosis of the interstitium **D**. Scale bars: 50 µm

During the management, screening for causes of secondary hypertension was performed. Her serum cortisol and catecholamine levels were slightly increased but did not meet the diagnostic criteria for Cushing’s disease or pheochromocytoma. Levels of thyroid-stimulating hormone and free thyroxine were also within the normal ranges. Polysomnography revealed severe obstructive sleep apnea syndrome with an apnea–hypopnea index of 52/h, which was probably caused by obesity (body mass index 31.4). This finding suggested that the MHT may have been partly caused by secondary hypertension resulting from obstructive sleep apnea. Continuous positive airway pressure therapy was started on hospital day 10. A nonselective β-blocker was started on day 15. A renal biopsy was performed on day 16 to rule out primary renal disease. Pathological examination revealed small occlusive (onion skin-like) lesions in the renal arteries (Fig. 2A–C). Extensive atrophic changes in the tubules and fibrosis of the interstitium were observed on Masson’s trichrome staining (Fig. 2D). Immunofluorescence analysis showed no significant deposition but electron microscopy showed scattered swollen endothelial cells, suggesting hypertension-related endothelial damage. These findings were compatible with malignant nephrosclerosis.

Her systolic BP remained above 130 mmHg, so spironolactone was added on hospital day 18, after which her BP and body weight gradually decreased (Fig. 1). She was discharged on day 25 with normal BP and improved renal function. Aliskiren and spironolactone were discontinued upon discharge because of hyperkalemia. Thereafter, her BP remained controlled. Her creatinine level showed a significant decrease to 1.14 mg/dL at 24 months after discharge (Fig. 1).

## Discussion

**Fig. 3:**
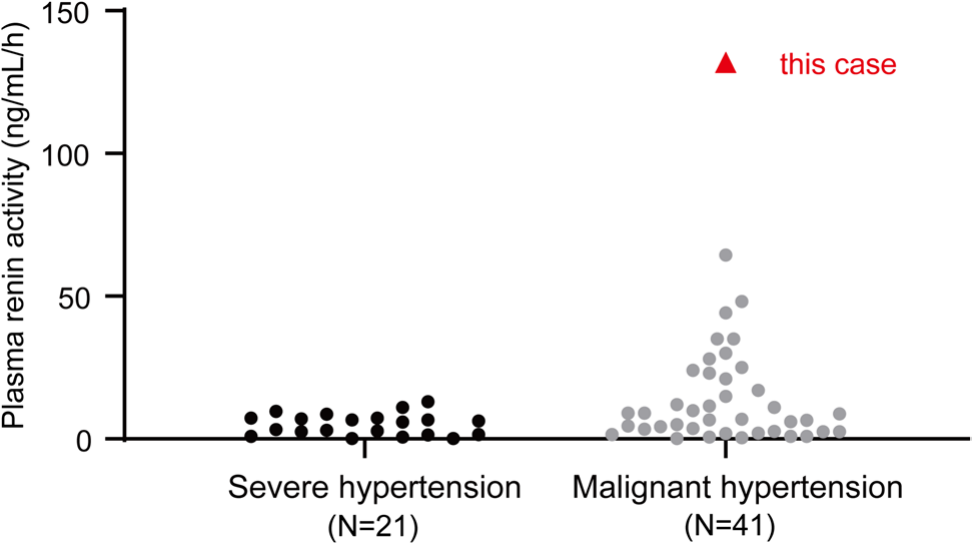
Plasma renin activity in patients with malignant hypertension and those with severe hypertension. Data for plasma renin activity were obtained from previous reports [1, 11, 13–15]. The present case is shown by a closed red triangle

We have encountered a case of MHT that presented as hypokalemia with hyperreninemia and hyperaldosteronism and acute renal deterioration caused by malignant nephrosclerosis. The primary mechanism for marked activation of the RAAS is juxtaglomerular ischemia resulting from intrarenal fibrinoid necrosis of small afferent arterioles and proliferative endarteritis of medium-sized and small arteries [12]. In patients with MHT, the pretreatment plasma renin activity (PRA) is strongly correlated with the creatinine level and the concentration of LDH, which is a marker of microangiopathy [1] and likely reflects vascular damage mediated via the downstream effects on angiotensin II and aldosterone. PRA was extremely high in our patient in comparison with previous reports on MHT [1, 11, 13–15] (Fig. 3). Furthermore, both LDH and creatinine were increased. These findings suggest that our patient had vascular damage and renal dysfunction that were severely accelerated by overactivation of the RAAS.

The 2023 European Society of Hypertension guidelines caution against rapid uncontrolled lowering of BP [16]. Therefore, to avoid a large and rapid reduction of BP, our patient was initially treated with a CCB rather than a RASI. In view of the patient’s marked hypertension, we performed magnetic resonance angiography to investigate the possibility of bilateral renal artery stenosis. After ruling out this condition, we initiated RASI therapy. However, since the patient’s BP remained high despite the maximum CCB dosage and the effect of a CCB on improvement of renal function is limited [4], an ARB/DRI combination was added on hospital day 4. An MRA was added on day 18, and the DRI and MRA were discontinued at discharge. The patient’s estimated glomerular filtration rate (eGFR) increased significantly from 10 mL/min/1.73 m^2^ at admission to 42 mL/min/1.73 m^2^ at 2 years after discharge. This finding is consistent with that of a recent retrospective study by Endo et al. that investigated the role of early initiation of RAAS blockade in 49 patients with hypertensive emergency [4]. They found that use of an ACEI or ARB was associated with a subsequent increase in eGFR, and severely diminished eGFR or massive proteinuria on admission had been associated with a reduced probability of renal survival. The recovery of renal function in our patient despite his severe renal failure and massive proteinuria suggests that RAAS blockade by combination therapy, which leads to a further reduction in angiotensin II and aldosterone, during the acute phase of MHT may be more beneficial than monotherapy in such patients.

Given that the PRA level is much higher in patients with MHT than in those with mild-to-moderate hypertension, compensatory activation of renin induced by ARB or ACEI monotherapy can make it difficult to suppress the angiotensin II type 1 receptor (AT1R) adequately in MHT. However, our patient had extremely high PRA on admission, which normalized almost 2 weeks after initiation of antihypertensive treatment. Although there are no reports showing that ARB or ACEI/DRI combination therapy has an effect on PRA in patients with MHT, it is possible that the clear reduction of PRA on a maximal dose of ARB in our case was primarily attributable to the effects of aliskiren. Indeed, PRA has been found to decrease on aliskiren but to increase on an ARB in both healthy volunteers [17] and patients with mild-to-moderate hypertension [18, 19]. Similarly, Case et al. showed that PRA increased after treatment with the ACEI captopril in patients with severe hypertension [13]. A recent retrospective study by Ueno et al. showed that PRA was clearly reduced by 150 mg/day of aliskiren in patients with hypertensive emergency and that strong suppression of PRA was correlated with improvement in eGFR [7]. Therefore, combining a DRI with an ARB or ACEI during the acute phase of MHT, taking care to avoid excessive hypotension and deterioration of renal function, may be beneficial because DRI can adequately suppress the AT1R by inhibiting compensatory activation of renin.

In the ALTITUDE study, hyperkalemia and hypotension were more common in patients with type 2 diabetes and kidney disease who were receiving an ARB or ACEI in combination with aliskiren than in those receiving an ARB or ACEI alone [5]. However, there were significant differences in the baseline plasma potassium and systolic BP values between our patient and participants in ALTITUDE. Since the effect of combination therapy on plasma potassium and BP were observed promptly in the ALTITUDE study, it is possible that the adverse effects of RAAS combination therapy may confer benefits during the early phase in patients with MHT. Imbalzano et al. demonstrated that adding aliskiren to optimal conventional therapy which already included an ACEI or an ARB achieved greater reductions in BP and urinary albumin excretion with preserved renal function [20]. Similarly, a reduction of the urinary albumin to creatinine ratio was observed in the ALTITUDE study [5]. Therefore, combination therapy of DRI with an ARB or ACEI might be beneficial in patients with uncontrolled hypertension, although closer monitoring of the serum potassium level is required. The similarity in beneficial effects between DRI/ACEI or ARB and ACEI/ARB suggests that monotherapy with a RASI might not completely block the AT1R in some patients with uncontrolled hypertension. It has been speculated that an ARB may induce a compensatory increase in PRA, which could limit its antihypertensive effects [21]. Although the precise mechanism via which combination therapy acts on the AT1R remains unknown, it is possible that adequate suppression of the AT1R could be achieved by combination therapy in patients with uncontrolled hypertension.

**Fig. 4:**
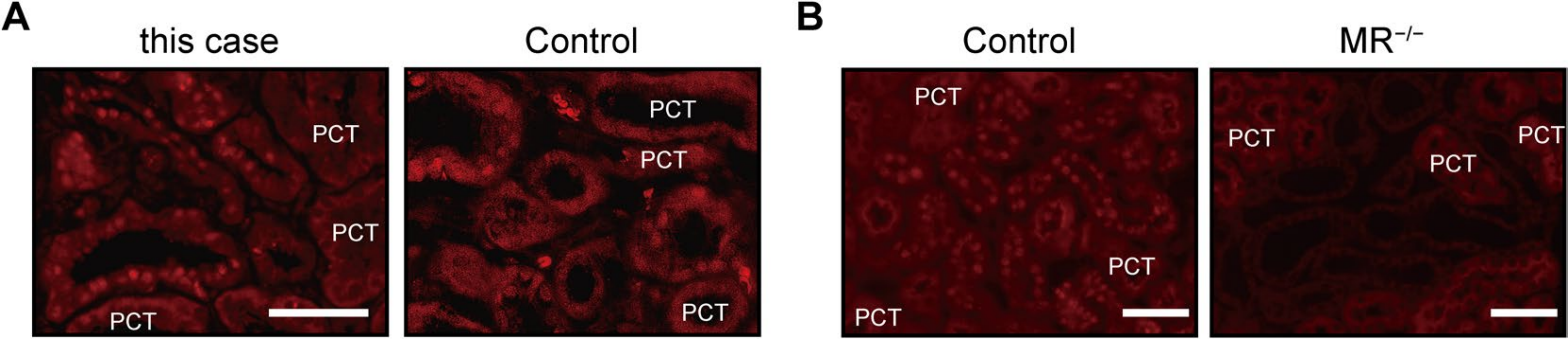
Mineralocorticoid receptor expression in this case. Immunofluorescence revealed that the mineralocorticoid receptor (MR) was localized to the nucleus along the distal nephron in our patient **A**. Anti-MR antibody was validated in MR^*−/−*^ mice showing that the nuclear MR signal along the distal nephron was clearly reduced in comparison with control mice, as previously reported [23] **B**. The anti-MR 6G1 antibody (#MABS496; EMD Millipore Corp, Billerica, MA) was used. Scale bars: 50 µm. PCT, proximal convoluted tubule

MRA agents directly downregulate the epithelial sodium channels along the distal convoluted tubule and through the collecting duct and indirectly downregulate the sodium chloride cotransporter along the distal convoluted tubule by increasing the plasma potassium level, leading to an increase in urinary sodium excretion and reduction of BP [22]. In our case, after the renal biopsy, an MRA was added to treatment with an ARB and DRI, which further reduced BP and body weight, although the plasma aldosterone concentration (PAC) had already decreased. Consistent with this effect of the MRA, immunofluorescence staining using the anti-mineralocorticoid receptor (MR) antibody, which has been validated in MR knockout mice [23], confirmed that the MR was still localized to the nucleus in our patient before administration of the MRA (Fig. 4). The MR translocates from the cytoplasm to the nucleus when activated by aldosterone, particularly along the aldosterone-sensitive distal nephron. Therefore, in our patient, we presume that MR was still activated by aldosterone despite administration of a DRI and ARB. Furthermore, we have previously demonstrated that treatment with an MRA decreases body weight and increases urinary sodium excretion in mice with complete aldosterone deficiency [23], suggesting that an MRA can induce natriuresis and decrease BP by inhibiting aldosterone-independent activation of the MR. Similarly, in patients with resistant hypertension, the BP response to spironolactone is only weakly correlated with the PAC [24], and many patients with low or normal aldosterone still respond to this agent [25]. Additionally, spironolactone clearly lowered BP in patients with MHT in whom both PRA and PAC were normal [8]. These findings suggest that MRA add-on therapy may be effective for lowering BP in patients with MHT, even when PAC is normal or low.

**Fig. 5:**
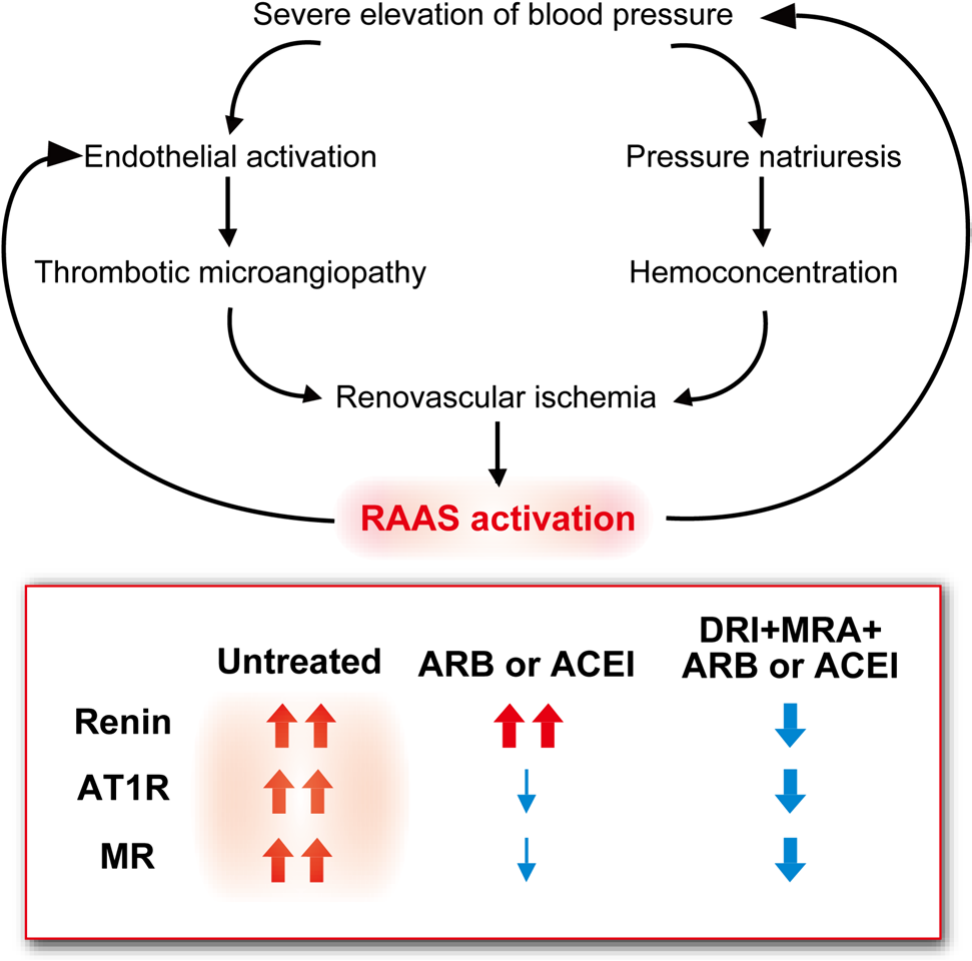
A combination of renin–angiotensin–aldosterone system inhibitors could potentially break the vicious cycle in malignant hypertension. *Top*, the vicious cycle of malignant hypertension (MHT) is caused by overactivation of the renin–angiotensin–aldosterone system (RAAS) [12]. *Bottom*, both the angiotensin II type 1 receptor (AT1R) and mineralocorticoid receptor (MR) are activated by the RAAS in the acute phase of MHT. Monotherapy with an angiotensin receptor blocker (ARB) or an angiotensin-converting enzyme inhibitor (ACEI) can induce compensatory activation of renin, which may make it difficult to achieve adequate suppression of the AT1R and MR. A direct renin inhibitor (DRI) can effectively suppress the AT1R by inhibiting compensatory activation of renin, while a mineralocorticoid receptor antagonist can decrease blood pressure by inhibiting aldosterone-dependent and independent activation of the MR. Combined therapy can adequately suppress the RAAS and break the vicious cycle of MHT

Considering that both an ARB and a DRI were administered simultaneously in this case and with uptitration of the ARB, the necessity for combined RASI therapy was unclear. However, the patient’s hypertension persisted despite use of a DRI and an ARB at maximum dose, suggesting that high-dose ARB monotherapy alone would likely have been insufficient. As shown in Table 1, there have been some reports on use of dual RASI therapy (ACEI + ARB or ARB + MRA) [8–11]. Interestingly, renal function was improved by this combination therapy in cases with hyperreninemia and hypokalemia but not in those with normoreninemia and normokalemia or hyperkalemia [8, 11]. In view of the similar improvement in renal function in response to triple RASI therapy in our case, combination therapy that includes an ARB, ACEI, MRA, and/or DRI, might be beneficial in patients with MHT and overactivation of the RAAS. Both aliskiren and spironolactone were discontinued before discharge due to hyperkalemia, and after discharge, BP remained well controlled, suggesting that such combination therapy may not be necessary during the chronic phase after breaking the vicious cycle of MHT and could potentially be harmful (Fig. 5).

**Table 1:** Reported cases of hypertensive emergency/malignant hypertension treated with a combination of renin–angiotensin–aldosterone system inhibitors

						On admission						Follow-up		
Author [ref]	Year	Age	Sex	Diagnosis	Therapy	BP (mmHg)	Cr (mg/dL)	uPCR (g/gCr)	K (mmol/L)	PRA (ng/mL/h)		Period (months)	Cr (mg/dL)	uPCR (g/gCr)
Tanemoto M [9]	2003	30	M	hypertensive emergency	ARB + ACEI	210/145	1.3	1.84	3.4	13.4		2/3	0.9	< 0.15
Tanemoto M [9]	2003	30	M	accelerated hypertension	ARB + ACEI	217/154	6.1	5.28	3.4	79		12	1.7	0.5
Tanemoto M [9]	2003	24	M	malignant hypertension	ARB + ACEI	269/140	3.2	2.27	2.7	52.7		2	1.6	0.5
Villafuerte L HM [8]	2018	38	M	malignant hypertension	ARB + MRA	250/160	8.83	2.5	7.1	Normal		1	(HD)	N/A
Watanabe K [10]	2022	44	F	hypertensive emergency	ARB + MRA	226/109	3.72	5.7	2.7	86.1		18	1.62	0.7
Sawamura M [11]	2023	37	M	malignant hypertension	ARB + MRA	240/140	4.16	2.95	4.2	2.7		28	4	N/A
**Present case**	2024	41	F	malignant hypertension	ARB + DRI + MRA	242/186	3.93	4	2.7	131.9		24	1.14	0.49

*ACEI* Angiotensin-converting enzyme inhibitor, *ARB* Angiotensin receptor blocker, *BP* Blood pressure, *Cr* Creatinine, *DRI* Direct renin inhibitor, *HD* hemodialysis, *MRA* Mineralocorticoid receptor antagonist, *PRA* Plasma renin activity, *uPCR* Urine protein creatinine ratio

In conclusion, we encountered a case of renal failure induced by MHT that was treated successfully using a combination of RASIs. A DRI can efficiently suppress the AT1R by inhibiting compensatory activation of renin, while an MRA can decrease BP by inhibiting both aldosterone-dependent and aldosterone-independent activation of the MR in patients with MHT (Fig. 5). Given that early initiation of RASIs is associated with a better renal outcome [4], it may be important to suppress the RAAS cascade adequately during the acute phase of MHT. This case supports the need for further studies evaluating the use of a combination of RASIs in patients with MHT.

## Data Availability

The datasets collected for this case report are available from the corresponding author on reasonable request.

## Abbreviations

*ACEI*: Angiotensin-converting enzyme inhibitor
*ARB*: Angiotensin receptor blocker
*AT1R*: Angiotensin II type 1 receptor
*BP*: Blood pressure
*CCB*: Calcium channel blocker
*DRI*: Direct renin inhibitor
*LDH*: Lactate dehydrogenase
*MHT*: Malignant hypertension
*MR*: Mineralocorticoid receptor
*MRA*: Mineralocorticoid receptor antagonist
*PAC*: Plasma aldosterone concentration
*PRA*: Plasma renin activity
*RAAS*: Renin–angiotensin–aldosterone system
*RASI*: Renin–angiotensin–aldosterone system inhibitor
